# Interferon-gamma improves impaired dentinogenic and immunosuppressive functions of irreversible pulpitis-derived human dental pulp stem cells

**DOI:** 10.1038/srep19286

**Published:** 2016-01-18

**Authors:** Soichiro Sonoda, Haruyoshi Yamaza, Lan Ma, Yosuke Tanaka, Erika Tomoda, Reona Aijima, Kazuaki Nonaka, Toshio Kukita, Songtao Shi, Fusanori Nishimura, Takayoshi Yamaza

**Affiliations:** 1 Department of Molecular Cell Biology and Oral Anatomy, Kyushu University Graduate School of Dental Science, Fukuoka, Japan; 2 Department of Periodontology, Kyushu University Graduate School of Dental Science, Fukuoka, Japan; 3 Department of Pediatric Dentistry, Kyushu University Graduate School of Dental Science, Fukuoka, Japan; 4 Department of Pediatric Dentistry, Guanghua School of Stomatology, Hospital of Stomatology, Sun Yat-sen University, Guangzhou, China; 5 Department of Histology and Neuroanatomy, Faculty of Medicine, Saga University, Saga, Japan; 6 Department of Anatomy and Cell Biology, School of Dental Medicine, University of Pennsylvania, PA, USA

## Abstract

Clinically, irreversible pulpitis is treated by the complete removal of pulp tissue followed by replacement with artificial materials. There is considered to be a high potential for autologous transplantation of human dental pulp stem cells (DPSCs) in endodontic treatment. The usefulness of DPSCs isolated from healthy teeth is limited. However, DPSCs isolated from diseased teeth with irreversible pulpitis (IP-DPSCs) are considered to be suitable for dentin/pulp regeneration. In this study, we examined the stem cell potency of IP-DPSCs. In comparison with healthy DPSCs, IP-DPSCs expressed lower colony-forming capacity, population-doubling rate, cell proliferation, multipotency, *in vivo* dentin regeneration, and immunosuppressive activity, suggesting that intact IP-DPSCs may be inadequate for dentin/pulp regeneration. Therefore, we attempted to improve the impaired *in vivo* dentin regeneration and *in vitro* immunosuppressive functions of IP-DPSCs to enable dentin/pulp regeneration. Interferon gamma (IFN-γ) treatment enhanced *in vivo* dentin regeneration and *in vitro* T cell suppression of IP-DPSCs, whereas treatment with tumor necrosis factor alpha did not. Therefore, these findings suggest that IFN-γ may be a feasible modulator to improve the functions of impaired IP-DPSCs, suggesting that autologous transplantation of IFN-γ-accelerated IP-DPSCs might be a promising new therapeutic strategy for dentin/pulp tissue engineering in future endodontic treatment.

The dentin/pulp complex does not self-remodel/regenerate, but forms reparative dentin in response to diverse tissue injury^1^,^2^. Tumor necrosis factor alpha (TNF-α) and interferon gamma (IFN-γ) are involved in the pathogenesis of dental pulpitis^3^,^4^, which can be clinically categorized as either reversible or irreversible pulpitis^5^. In irreversible pulpitis, the injured dental pulp tissue does not recover once the pathogen(s) is removed completely. Therefore, clinically, pulp tissue with irreversible pulpitis is completely removed and replaced by artificial materials such as cements and gutta percha. Teeth that receive endodontic treatment lose their physiological bioactivity, including strength, sensitivity and immune defense, and many ultimately require extraction because of fractures or caries. Therefore, regeneration of the bioactive dentin/pulp complex is considered an ideal endodontic therapy for pulpectomized teeth.

Dental pulp stem cells (DPSCs) have been identified in the healthy dental pulp tissue of human impacted third molars^6^, and are regarded as a subpopulation of mesenchymal stem cells (MSCs). Recent investigation of DPSCs has discovered various stem cell properties, including self-renewal, multipotency into odontoblasts, chondrocytes and adipocytes, an *in vivo* regenerative capacity of the dentin/pulp complex, heterogeneity, and immunomodulatory functions^6^,^7^,^8^,^9^. Based on these unique properties of DPSCs, healthy dental pulp tissue has been considered a promising resource for pulp regeneration^10^.

Patient-derived pulpectomized pulp tissue is also considered to be a feasible and ideal source for DPSC-based pulp regeneration because of its dentinogenic capacity^11^,^12^. Although recent studies have attempted to isolate and characterize stem cells from inflamed dental pulp tissue that is clinically diagnosed with irreversible pulpitis^11^,^12^,^13^,^14^, many properties of pulpitis-derived DPSCs remain unclear. Recently, pulpitis-derived DPSCs have been shown to exhibit less efficacy for dental pulp regeneration and T cell immunosuppression^13^,^14^. However, a practical approach to improving the deficient functions of pulpitis-derived DPSCs has not been revealed.

In this study, to clarify the properties of pulpitis-derived DPSCs, we isolated stem cells from human dental pulp tissue with irreversible pulpitis, referred to as IP-DPSCs, using colony-forming unit-fibroblasts (CFU-Fs)^15^, and determined a variety of MSC properties including clonogenicity, self-renewal capacity, multidifferentiation ability into odontoblasts, adipocytes, endothelial cells and neural cells, *in vivo* dentin regenerative capacity, heterogeneity, and immunomodulatory functions. Furthermore, we attempted to develop an *ex vivo* approach to improve IP-DPSCs by treatment with TNF-α and IFN-γ.

## Results

### Stemness of IP-DPSCs

**Figure 1:**
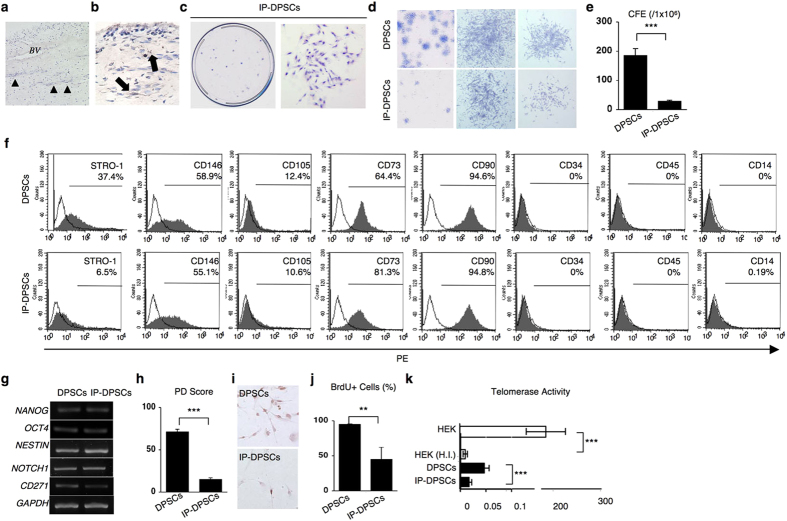
Characterization of stem cells isolated from inflamed human dental pulp. (**a**) Histochemical images of irreversible pulpitis tissue. H&E staining. BV: blood vessel. Arrowheads: nerve fibers. (**b**) Immunohistochemical localization of STRO-1-positive cells (arrows) in irreversible pulpitis tissue. Hematoxylin staining. (**c–e**) Capability of CFU-Fs of IP-DPSCs. Toluidine blue staining. Representative image of CFU-Fs on a culture dish (**c left**) and fibroblastic colony-forming cells (**c right**). Differences in colony size and density (**d**). Colony-forming efficiency (CFE) per 1 × 10^6^ cells (**e**). (**f**) Immunophenotype assay by flow cytometric analysis. White area: histograms stained with control antibody; grey area: histograms stained with antibodies against cell surface antigens. Percentiles indicate the average of each antigen. PE: phycoerythrin. (**g**) Gene expression for embryonic stem and neural crest cell markers. Semi-quantitative RT-PCR assay. OCT4: octamer 4, GAPDH: glyceraldehyde 3-phosphate dehydrogenase. (**h**) Population-doubling assay. (**i,j**) Cell proliferation analysis by BrdU incorporation assay. Representative images of BrdU-positive cells. Hematoxylin staining (**i**). Percentiles of BrdU-positive nuclei to the total nuclear cells (**j**). (**k**) Telomerase activity test. TRAP-qPCR assay. HEK: Human Embryonic Kidney 293 cells, H.I. heat-inactivated treatment. (**e,f,h,j,k)**: n = 3 per group. **P < 0.01 and ***P < 0.005. Graph bars show the means ± s.e.m.

Histological analysis demonstrated that inflamed dental pulp tissue freshly obtained from teeth that were clinically diagnosed with irreversible pulpitis consisted of dense connective tissue supplied with blood vessels and nerve fibers (Fig. 1a). An early MSC marker, STRO-1, was detected on cells in the inflamed pulp tissue (Fig. 1b), suggesting that inflamed dental pulp tissue may contain MSCs, as reported in healthy human dental pulp tissue^16^.

DPSCs isolated from clinically healthy dental pulp tissue, referred to as healthy DPSCs, can be shared with MSC properties, including clonogenicity, self-renewal, MSC surface antigens, multidifferentiation, *in vivo* dentin regeneration, and immunosuppression^6^,^7^,^9^. However, the properties of DPSCs isolated from irreversible pulpitis tissue, IP-DPSCs, have not yet been fully revealed^11^,^12^,^14^. To closely examine the properties of IP-DPSCs, at the beginning of this study, cells were isolated from fresh irreversible pulpitis tissue with a standard CFU-F method^15^. Single cells were independently attached to the plastic culture dishes and then formed cell clusters and CFU-Fs (Fig. 1c) with different sizes and varied density (Fig. 1d), but the IP-DPSCs showed significantly lower colony-forming efficacy when compared with healthy DPSCs (Fig. 1e). Flow cytometric analysis demonstrated that IP-DPSCs were positive to STRO-1, CD146, CD105, CD73, and CD90, but negative to hematopoietic cell markers CD34, CD45, and CD14, as seen in healthy DPSCs (Fig. 1f). Reverse transcription polymerase chain reaction (RT-PCR) demonstrated that IP-DPSCs expressed genes for embryonic stem cells, NANOG and octamer 4, and for neural crest cells, NESTIN, NOTCH1, and CD271 (Fig. 1g). IP-DPSCs expressed a markedly reduced CD271 when compared with healthy DPSCs. By population-doubling and bromodeoxyuridine (BrdU) assays, IP-DPSCs showed a significantly suppressed proliferation capacity when compared with healthy DPSCs (Fig. 1h–j). IP-DPSCs expressed lower telomerase activity than healthy DPSCs (Fig. 1k). Therefore, these data suggested that IP-DPSCs retained stemness as MSCs, but expressed different essential characteristics when compared with healthy DPSCs.

### Multipotency of IP-DPSCs

**Figure 2:**
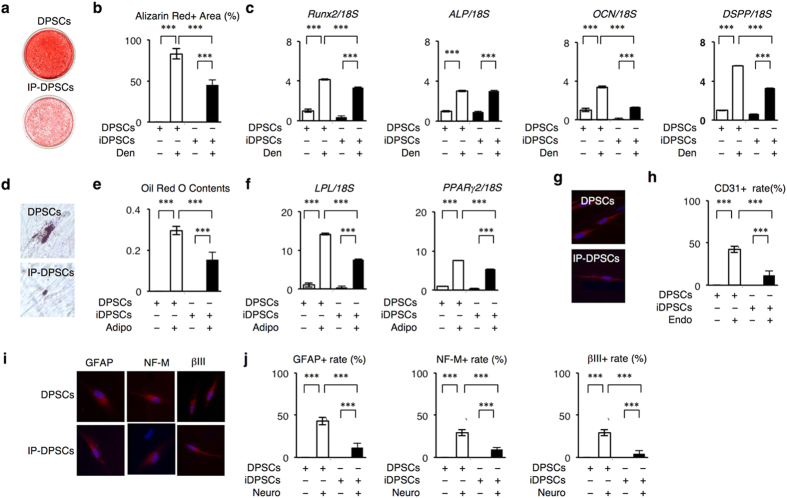
Multi-lineage differentiation capacity of IP-DPSCs. (**a–c**) *In vitro* dentinogenic capacity (Den). Representative images of Alizarin Red staining (**a**). Percentiles of Alizarin Red-positive area to the total area (**b**). Ratio of odontoblast-specific genes to 18S rRNA (18S). RT-qPCR (**c**). (**d–f**) *In vitro* adipogenic capacity (Adipo). Representative images of Oil Red O staining (**d**). Measurement of Oil Red O content (**e**). Ratio of adipocyte-specific genes to 18S rRNA. RT-qPCR (**e**). (**g,h**) *In vitro* endothelial cell differentiation capacity (Endo). Immunofluorescent images of CD31-positive cells. DAPI staining (**g**). Percentiles of CD31-positive cells to total cells (**h**). (**i,j**) *In vitro* neural cell differentiation capacity (Neuro). Immunofluorescent images of neural marker-positive cells. DAPI staining (**i**). Percentiles of neural marker-positive cells to total cells (**j**). (**b,c,e,f,h,j)**: n = 3 per group. Relative results to non-induced DPSC group. ***P < 0.005. Graph bars show the means ± s.e.m.

When IP-DPSCs were cultured under a dentinogenic condition for 4 weeks, they were capable of forming calcium-deposited nodules positive to Alizarin Red staining (Fig. 2a,b). RT-PCR and quantitative RT-PCR (RT-qPCR) demonstrated that IP-DPSCs at 1 week after the induction expressed odontoblast-specific genes including runt-related gene 2 (Runx2), alkaline phosphatase (ALP), osteocalcin (OCN), and dentin sialophosphoprotein (DSPP) (Fig. 2c, Supplementary Figure 1a) and showed ALP activity (data not shown). IP-DPSCs were then cultured under an adipogenic condition for 6 weeks. Oil Red O staining assay demonstrated lipid accumulation in IP-DPSCs (Fig. 2d,e). RT-PCR and RT-qPCR confirmed the expression of adipocyte-specific genes, including lipoprotein lipase (LPL) and peroxisome proliferator-activated receptor γ2 (PPARγ2) (Fig. 2f, Supplementary Figure 1b). Immunofluorescence showed the expression of CD31 on IP-DPSCs that were cultured under an endothelial cell differentiation condition for seven days (Fig. 2g,h). We also treated IP-DPSCs under a neural cell induction condition for 7 days. Immunofluorescent analysis detected the expression of glial fibrillary acidic protein (GFAP), neurofilament M (NF-M), and tubulin βIII (βIII) on IP-DPSCs (Fig. 2i,j). IP-DPSCs also showed a reduced capability to differentiate into odontoblasts, adipocytes, endothelial cells, and neural cells in comparison with healthy DPSCs (Fig. 2).

### *In vivo* dentin/pulp complex regeneration of IP-DPSCs

**Figure 3:**
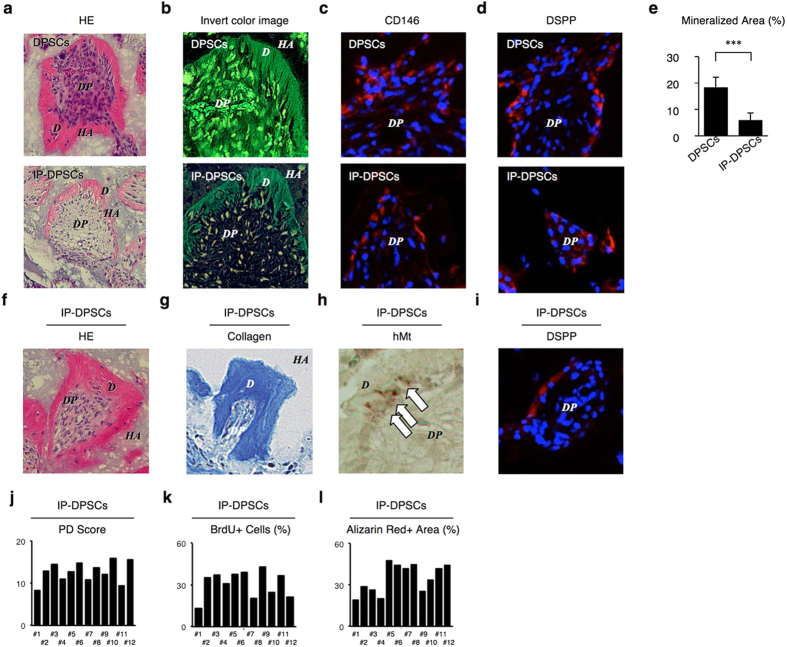
*In vivo* dentinogenic and self-renewal capacities and heterogeneity of IP-DPSCs. (**a–e**) *In vivo* dentinogenic capacity assay. Representative images of transplants. H&E staining (**a**). Invert color images of H&E images (**b**). (**c,d**) Immunofluorescent localization of human CD146 (**c**) and DSPP (**d**). DAPI staining (**c,d**). (**a–d)**: DSPP *D*: dentin, *DP*: dental pulp, *HA*: HA/TCP carrier. Percentiles of *de novo* dentin area to the total area. n = 3 per group. ***P < 0.005. Graph bars show the means ± s.e.m (**e**). (**f–i**) Self-renewal assay. Representative image of secondary transplants. H&E staining (**f**). Alinine blue collagen stainig (**g**). Immunohistochemical localization of human mitochondria (hMt)-positive cells (arrows). Hematoxylin staining (**h**). Immunofluorescent localization of DSPP. DAPI staining (**i**). (**j–l**) Heterogeneity. Single colony-derived cell assay. Population-doubling assay (**j**). Percentiles of BrdU-positive nuclei to total nuclear cells (**k**). Percentiles of Alizarin Red-positive area to the total area (**l**).

IP-DPSCs were subcutaneously implanted with hydroxyapatite/tricalcium phosphate (HA/TCP) carriers under the dorsal skin of immunocompromised BALB/c nu/nu mice and left in place for 8 weeks (Supplementary Figure 2). Histological analysis showed that dentin/pulp complex-like structures were found in the implant tissues (Fig. 3a,b). This was similar to the findings of previous studies^6^,^7^. Immunofluorescence showed that human CD146- or DSPP-positive cells were arranged along the surface of the *de novo* mineralized matrix (Fig. 3c,d). In comparison, control transplants that received implantation of HA/TCP alone (without IP-DPSCs) did not show any mineralized tissue or human CD146 antibody-positive cells (data not shown). However, IP-DPSCs exhibited a reduced capacity for *in vivo* dentin/pulp complex regeneration in comparison with healthy DPSCs (Fig. 3e).

### Self-renewal capability of IP-DPSCs

Sequential transplantation is the traditional and gold standard method to determine the self-renewal capacity of stem cells, including DPSCs^6^,^7^,^8^ (Supplementary Figure 2). IP-DPSCs were first transplanted with HA/TCP placed under the dorsal skin of immunocompromised mice. Cells were isolated from the primary transplants 8 weeks after the primary implantation, and were then transplanted under the dorsal skin of different immunocompromised mice for a further 8 weeks. Histological investigation revealed that *de novo* structures in the secondary transplants expressed dentin/pulp complex-like structures in the primary transplants (Fig. 3f,g). Human mitochondria- or DSPP-positive cells were arranged on the *de novo* mineralized matrix in the secondary transplants (Fig. 3h,i). Population-doubling (Fig. 1h) and telomerase activity (Fig. 1k) in IP-DPSCs were associated with a self-renewal potential of stem cells^17^. Collectively, these results verified that IP-DPSCs have a self-renewal capacity.

### Heterogeneity of IP-DPSCs

Heterogeneity in MSCs^18^,^19^ is one of the unique characteristics of healthy DPSCs^7^. To examine heterogeneity in IP-DPSCs, a total of 12 clonogenic single colonies were obtained from irreversible pulpitis tissue. Each single colony-derived cells expressed a different population-doubling score (Fig. 3j), cell proliferation capacity (Fig. 3k), and *in vitro* calcium deposition (Fig. 3l). These findings indicated that IP-DPSCs were a heterogeneous population.

### Immunosuppressive function of IP-DPSCs

**Figure 4:**
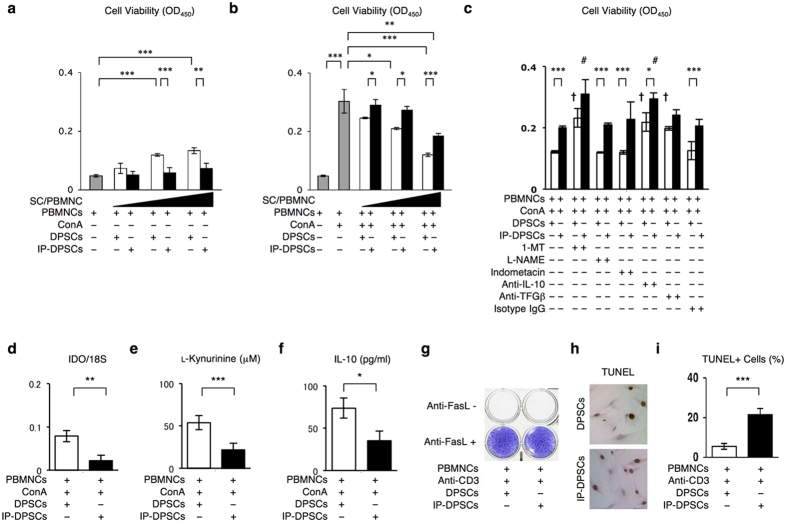
Immunomodulatory effects of IP-DPSCs. (**a–c**) Cell viability of human PBMNCs cultured with different ratios of DPSCs (SC) (SC/PBMNC = 0.01, 0.1, and 1) in the absence (**a**) or presence (**b**) of ConA. Co-culture of PBMNCs with DPSCs (SC/PBMNC = 1) under ConA stimulation with varied inhibitors and neutralized antibodies. Anti-IL10: anti-IL-10 antibody, Anti-TGFβ: anti-TGF-β antibody (**c**). (**d**) Expression of IDO in DPSCs. RT-qPCR assay. Ratio of IDO mRNA to 18S rRNA (18S). (**e**) Expression of L-kynurenine in culture supernatants of DPSCs. (**f**) Expression of IL-10 in culture supernatants of DPSCs. ELISA. (**g**–**i**) Cell death assay of DPSCs co-cultured with anti-CD3 antibody (Anti-CD3)-activated PBMNCs for 3 days (**g**) or 1 day (**h,i**). Anti-FasL: anti-FasL antibody. Toluidine blue staining (**g**). TUNEL assay (**h,i**). Representative images of TUNEL-positive cells (**h**). Percentiles of TUNEL-positive cells to total nuclear cells (**i**). (**a–f,i**): n = 3 per group. *P < 0.05, **P < 0.01, and ***P < 0.005. Graph bars show the means ± s.e.m. **c**: † < 0.05 vs. culture of PBMNCs with DPSCs in the absence of inhibitors and antibodies, ^#^*P* < 0.05 vs. culture of PBMNCs with DPSCs in the absence of inhibitors and antibodies.

Healthy DPSCs exhibit T cell suppression^9^. Different numbers of γ-irradiated IP-DPSCs were directly co-cultured with human peripheral blood mononuclear cells (PBMNCs) stimulated with or without concanavalin A (ConA) (10 μg/ml) (Supplementary Figure 3a). Under a ConA-free condition, any number of IP-DPSCs did not affect the cell viability of PBMNCs (Fig. 4a). In comparison, under a ConA-stimulated condition, the PBMNC viability was suppressed depending on the cell number of IP-DPSCs (Fig. 4b). However, IP-DPSCs exhibited a reduced suppressive function in response to ConA-activated PBMNCs when compared with healthy DPSCs (Fig. 4b). Next, PBMSCs were co-cultured indirectly with IP-DPSCs or healthy DPSCs in a transwell culture system (Supplementary Figure 3). Under concanavalin A (ConA)-free condition, PBMNCs exhibited similar cell viability between direct and indirect co-culture systems (Supplementary Figure 4a). However, under a ConA-stimulated condition, the PBMNC viability did not showed any reduction depending on the cell number of IP-DPSCs in the traswell system (Supplementary Figure 4b).

Direct contact of MSCs with T cells regulates immune tolerance through the Fas/Fas ligand (FasL) pathway^20^. On the other hand, many soluble factors secreted from MSCs participate in the immunosuppression of T cells^21^,^22^,^23^,^24^. In this study, the co-culture of ConA-stimulated PBMNCs with IP-DPSCs or healthy DPSCs was treated with 1-methyl-L-tryptophan (1-MT) (indoleamine 2,3-dioxygenase [IDO] inhibitor), N-nitro-L-arginine methyl ester (L-NAME) (nitric oxide synthase [NOS] inhibitor), indomethacin (cyclooxygenase [COX] inhibitor), and antibodies neutralized to IL-10 and transforming growth factor (TGF)-β (Supplementary Figure 5a). L-NAME, indomethacin, and anti-TGF-β antibody did not recover the suppressed PBMNC viability when co-cultured with IP-DPSCs or healthy DPSCs (Fig. 4c). Treatment with 1-MT or anti-IL-10 antibody effectively enabled the IP-DPSC- or healthy DPSC-mediated inhibition of ConA-activated PBMNC viability (Fig. 4c). RT-qPCR analysis demonstrated that IP-DPSCs expressed lower IDO mRNA than healthy DPSCs when co-cultured with ConA-stimulated PBMNCs (Fig. 4d). The IP-DPSC co-culture produced less L-kynurenine as a result of IDO catabolism than the healthy DPSC co-culture (Fig. 4e). Enzyme-linked immunosorbent assay (ELISA) showed that the IP-DPSC co-culture group secreted lower human IL-10 in the conditioned medium (CM) of the co-cultures when compared with the healthy DPSC co-culture group (Fig. 4f). These results suggested that IP-DPSCs retained immunosuppressive function, but IP-DPSCs expressed reduced immunosuppression in comparison with healthy DPSCs.

### Cell survival of IP-DPSCs

Activated T cells induce apoptosis of MSCs through the Fas/FasL pathway^25^,^26^. When IP-DPSCs and healthy DPSCs were co-cultured with anti-CD3 antibody-activated PBMNCs for 3 days (Supplementary Figure 5b), the IP-DPSCs were also induced into cell death (Fig. 4g). However, the treatment with anti-Fas antibody inhibited cell death of the IP-DPSCs (Fig. 4g). The activated PBMNCs induced terminal deoxynucleotidyl transferase dUTP nick end labeling (TUNEL)-positive cells in IP-DPSCs one day after the co-culture (Fig. 4h). The number of TUNEL-positive cells was greater in IP-DPSCs than in healthy DPSCs (Fig. 4i).

### IFN-γ treatment improved dentinogenic dysfunction of IP-DPSCs

**Figure 5:**
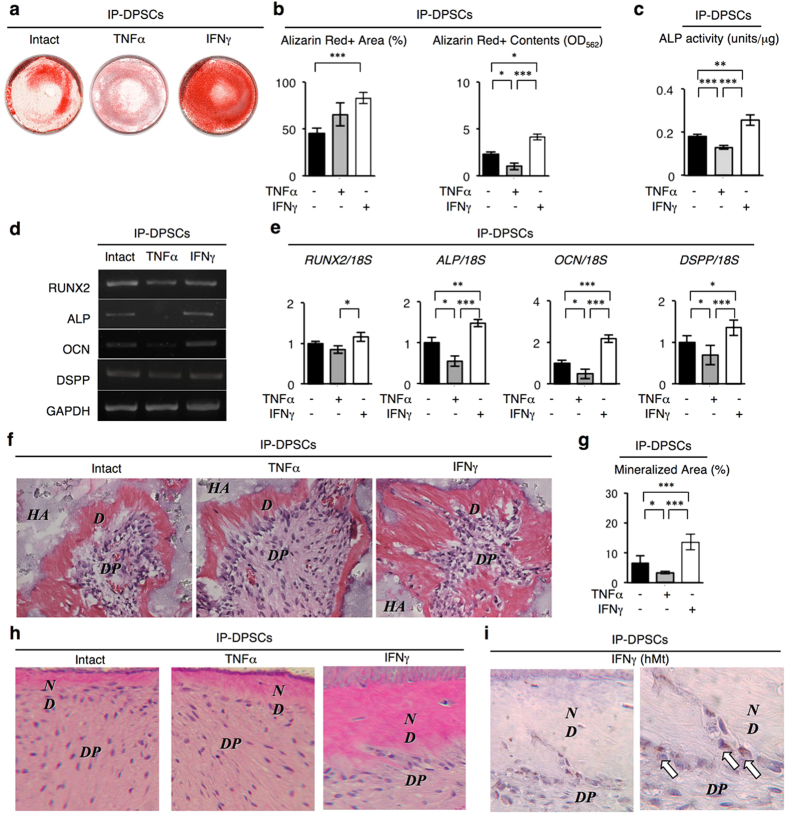
Effects of IFN-γ and TNF-α on dentinogenic capacity of IP-DPSCs. (**a–e**) *In vitro* dentinogenic capacity. Representative images of Alizarin Red staining (**a**). Percentiles of Alizarin Red-positive area to the total area (**b**). ALP activity assay (**c**). Semi-quantitative RT-PCR for odontoblast-specific genes (**d**). Ratio of odontoblast-specific genes to 18S rRNA (18S). RT-qPCR Relative results to non-treated (intact) IP-DPSC group. (**e**). (**f,g**) *In vivo* dentinogenic capacity. Representative images of transplants. *D*: dentin, *DP*: dental pulp, *HA*: HA/TCP carrier (**f**). Percentiles of *de novo* dentin area to the total area (**g**). (**h,i**) *In vivo* dentin regeneration on human dentin. *DP*: dental pulp, *ND*: newly formed dentin. H&E staining (**h**). Immunohistochemical localization of hMt-positive cells (arrows). Hematoxylin staining (**i**). (**b,c,e,g**): n = 3 per group. *P < 0.05, **P < 0.01, and ***P < 0.005. Graph bars show the means ± s.e.m.

TNF-α and IFN-γ have been known to participate in the immunomodulatory and dentinogenic functions of healthy DPSCs^9^,^13^. Here, we investigated whether TNF-α or IFN-γ improve the *in vitro* or *in vivo* impaired dentin formation of IP-DPSCs. IFN-γ-stimulated IP-DPSCs were capable of forming a significant number of calcium-deposited nodules positive to Alizarin Red staining 4 weeks after the induction (Fig. 5a,b). IFN-γ-stimulated IP-DPSCs showed higher ALP activity (Fig. 5c) and markedly upregulated the expression of odontoblast/osteoblast-specific genes, including Runx2, ALP, osteocalcin, and DSPP (Fig. 5d,e) 1 week after the induction. On the other hand, TNF-α-treated IP-DPSCs showed a markedly reduced dentinogenic capacity *in vitro* when compared with non-stimulated (intact) and IFN-γ-stimulated IP-DPSCs (Fig. 5a–e).

Next, TNF-α- or IFN-γ-pretreated IP-DPSCs were subcutaneously transplanted with HA/TCP into immunocompromised mice. Histological analysis demonstrated that IFN-γ-treatment enhanced the *in vivo* capability of dentin/pulp complex-like structures in the implant tissues (Fig. 5f,g). On the other hand, TNF-α-treatment reduced the *in vivo* capability (Fig. 5f,g). To further examine the effect of TNF-α and IFN-γ on IP-DPSC-mediated dentin/pulp regeneration, TNF-α and IFN-γ-treated IP-DPSCs were transplanted into human tooth root canals (Supplementary Figure 6). The IP-DPSCs formed a dentin-like structure directly on the surface of existing human tooth root dentin (Fig. 5h). IFN-γ-treatment abundantly deposited *de novo* dentin on a pre-existing human dentin surface, whereas TNF-α treatment did not (Fig. 5h). The newly formed dentin-like structure did not contain dentinal tubules, and the charged cells arranged on its surface and embedded within it. Immunohistochemical assay with anti-human mitochondria antibody revealed that the lining and embedded cells were of human origin (Fig. 5i). These structural findings indicated that IP-DPSC-mediated dentin/pulp complex regeneration on human dentin may be controlled under a similar mechanism as reparative dentin formation occurring in the physiological human dentin/pulp system, suggesting that IFN-γ treatment improved the impaired dentinogenic function of IP-DPSCs.

### IFN-γ treatment improved the impaired immunosuppressive efficacy of IP-DPSCs

**Figure 6:**
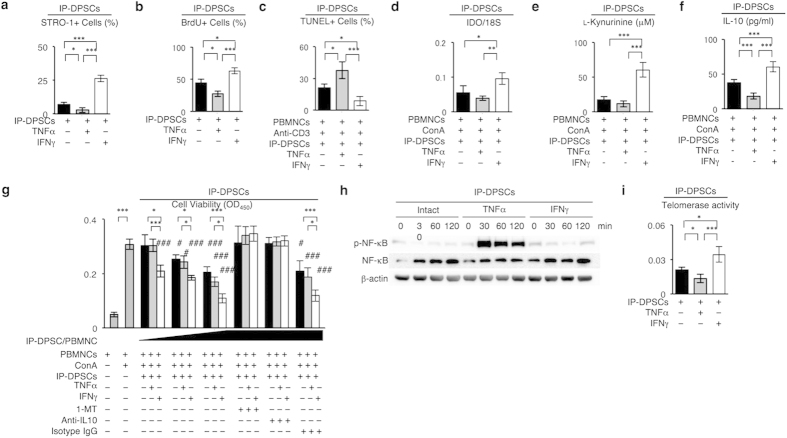
Effects of IFN-γ and TNF-α on STRO-1 expression, cell proliferation, cell death, and immunomodulatory function of IP-DPSCs. (**a**) Expression of STRO-1. Flow cytometry. (**b**) Percentiles of BrdU-positive nuclei to total nuclear cells. (**c**) Percentiles of TUNEL-positive cells to total nuclear cells. (**d**) Expression of IDO mRNA. RT-qPCR. Ratio of IDO to 18S rRNA (18S). (**e**) L-kynurenine in culture supernatants. (**f**) IL-10 in culture supernatants. ELISA. (**g**) Cell viability of human PBMNCs co-cultured with different ratios of IP-DPSCs (IP-DPSC/PBMNC = 0.01, 0.1, and 1). (**h**) Time-dependent expression of NF-κB. Western blotting. p-NF-κB: phosphorylated NF-κB. (**i**) Telomerase activity test. (**a–g,i)**: n = 3 per group. *P < 0.05, **P < 0.01, and ***P < 0.005. Graph bars show the means ± s.e.m. F: ^#^*P* < 0.05, ^###^*P* < 0.005 vs. culture of PBMNCs with DPSCs in the absence of inhibitors and antibodies.

We investigated the effect of TNF-α and IFN-γ treatment on STRO-1 expression. Flow cytometric analysis showed that TNF-α treatment significantly downregulated STRO-1 expression of IP-DPSCs, whereas IFN-γ treatment significantly upregulated STRO-1 expression, when compared with intact IP-DPSCs (Fig. 6a). BrdU incorporation assay demonstrated that TNF-α treatment markedly reduced the cell proliferation capacity of IP-DPSCs, whereas IFN-γ treatment significantly increased the IP-DPSC proliferation capacity, when compared with intact IP-DPSCs (Fig. 6b). Using the TUNEL method and under co-culture with anti-CD3 antibody-activated PBMNCs, TNF-α pretreatment markedly enhanced TUNEL-positive IP-DPSCs, whereas IFN-γ pretreatment suppressed TUNEL-positive IP-DPSCs, when compared with intact IP-DPSCs (Fig. 6c).

Next, we examined the effects of TNF-α and IFN-γ on the immunosuppressive functions of IP-DPSCs under co-culture with ConA-stimulated PBMNCs. RT-qPCR analysis and L-kynurenine production assay demonstrated that IFN-γ enhanced IDO mRNA expression and L-kynurenine production in IP-DPSCs co-cultured with ConA-stimulated PBMNCs, whereas TNF-α did not (Fig. 6d,e). Similarly, ELISA showed that IFN-γ induced IL-10 production, whereas TNF-α did not (Fig. 6f). We then co-cultured a greater number of IP-DPSCs with ConA-stimulated PBMNCs under treatment with TNF-α and IFN-γ. The cell viability of the PBMNCs was markedly suppressed when the IP-DPSCs were under treatment with TNF-α or IFN-γ (Fig. 6g). IFN-γ-mediated suppression was more effective than TNF-α-mediated inhibition (Fig. 6g). PBMNC suppression through TNF-α- and IFN-γ-stimulated IP-DPSCs was significantly inhibited by 1-MT and anti-IL-10 antibody treatments (Fig. 6g). These findings suggested that IFN-γ treatment may restore the impaired immunoregulatory function of IP-DPSCs.

We then investigated the time-dependent expressional change of nuclear factor kappa-light-chain-enhancer of activated B cells (NF-κB) in IP-DPSCs after TNF-α and IFN-γ treatment. TNF-α-treated IP-DPSCs expressed enhanced phosphorylation of NF-κB, whereas IFN-γ-treated and non-treated IP-DPSCs did not (Fig. 6h). We also examined the effects of TNF-α and IFN-γ on telomerase activity in IP-DSPCs. TNF-α markedly reduced the telomerase activity of IP-DPSCs (Fig. 6i). In comparison, IFN-γ significantly enhanced the telomerase activity of IP-DPSCs, when compared with non-treated IP-DPSCs (Fig. 6i).

## Discussion

The present isolation approach reveals the existence of CFU-F clonal populations^15^ in human dental pulp tissues diagnosed with irreversible pulpitis. The isolated CFU-Fs also express multipotency and immunological features as MSCs. Moreover, the present sequential transplantation and single colony assays demonstrated that the isolated CFU-Fs express an *in vivo* dentin/pulp complex regeneration ability, self-renewal capacity, and heterogeneity. Overall, these findings indicate that the present CFU-Fs isolated from irreversible pulpitis tissue display stem cell characteristics similar to MSCs and healthy DPSCs^6^,^7^,^8^. Furthermore, IP-DPSCs also demonstrated an immunosuppressive function similar to healthy DPSCs^9^. However, when compared with healthy DPSCs, the IP-DPSCs showed impaired colony-forming capacity, cell proliferation rate, multipotency, *in vivo* dentin regeneration and immunosuppressive functions.

Recent tissue engineering technology has shown that healthy DPSCs reconstruct a dentin/pulp-like structure on human dentin in a xenograft system using immunocompromised mice^8^,^27^. In dogs, autologous transplantation of DPSCs from freshly extracted teeth successively regenerates a vascularized dental pulp tissue in pulpectomized teeth^28^. The currently innovated preclinical grade of healthy human DPSCs combined with granulocyte colony-stimulating factor suggests that autologous healthy DPSC-based therapy may almost be ready for clinical application^29^,^30^. However, the opportunity to obtain healthy donor teeth is quietly limited at a general clinical situation. Therefore, IP-DPSCs isolated from diseased teeth might be a feasible source for dentin/pulp complex regeneration^11^,^12^. The present IFN-γ-mediated IP-DPSCs facilitated the regeneration of dysfunctional dentin/pulp and the immunosuppressive function of IP-DPSCs will aid in the development of autologous DPSC-based endodontic therapy.

Inflammatory pulp reactions including reversible and irreversible pulpitis are carried out by several etiologies/stimuli^5^. Human dental pulp tissue with irreversible pulpitis expresses high levels of TNF-α and IFN-γ^4^. Major inflammatory helper T cell cytokines, TNF-α and IFN-γ, have been known to affect the immunomodulatory and dentinogenic functions of healthy DPSCs^9^,^13^,^31^,^32^,^33^,^34^. However, the effects of TNF-α and IFN-γ on the immunomodulatory and dentinogenic capabilities of IP-DSPCs have not yet been clarified. Recent studies of short-term TNF-α exposure-enhanced dentinogenic activity in healthy DPSCs suggest that reparative dentin formation occurs in response to reversible pulpitis^32^,^35^,^36^. Meanwhile, prolonged and heavy exposure of TNF-α diminishes the mineralization potential of IP-DPSCs and healthy DPSCs^13^,^37^. In comparison, IFN-γ accelerates the osteoblastic differentiation of human MSCs *in vitro* and *in vivo*^38^. IFN-γ-treated bone marrow-derived mesenchymal stem cells (BMMSCs) and DPSCs also accelerated IDO production to enhance the suppressive effect on T cell proliferation^9^,^39^,^40^. The present IFN-γ treatment successfully recovered the dysfunction of IP-DPSC-mediated dentin regeneration *in vitro* and *in vivo* and the suppression of T cells via IDO, whereas TNF-α treatment did not. These data suggest that exogenous IFN-γ treatment to IP-DPSCs is a novel approach to improve its ability of dentin/pulp complex regeneration and immunosuppression.

BMMSCs derived from patients with systemic lupus erythematosus, which have impaired bone forming potential, express enhanced phosphorylation of NF-κB under accelerated TNF-α signaling^41^. The inhibition of NF-κB activation induces the osteogenic capacity of normal BMMSCs^42^. In healthy DPSCs, suppression of activated NF-κB also enhances odontogenic ability under TNF-α stimulation^31^,^43^. In the present study, TNF-α treatment caused NF-κB activation with improved dentin/pulp complex regeneration and T cell immunosuppression in IP-DPSCs^13^,^14^. On the other hand, IFN-γ treatment enhanced the ability for IP-DPSCs by activation of NF-κB independently. These findings suggest that a practical control for NF-κB activation may enable regulation of the impaired functions of IP-DPSCs, including dentin/pulp regeneration and T cell immunosuppression.

The ectopic human telomerase reverse transcriptase (TERT) gene in human BMMSCs induces bone formation *in vitro* and *in vivo* by enhancing a critical master transcription factor for osteoblast differentiation, Runx2^44^,^45^. TERT modulates FasL expression on BMMSCs to induce T cell apoptosis^46^. Recently, the non-steroidal anti-inflammatory drug aspirin (acetylsalicylic acid) has been shown to enhance telomerase activity and stimulate bone formation *in vitro* and *in vivo* as well as induce T cell apoptosis in BMMSCs and stem cells derived from human deciduous teeth^25^,^47^,^48^. In this study, IFN-γ-treated telomerase activity in IP-DPSCs facilitated *in vitro* and *in vivo* dentin formation and T cell suppression, whereas TNF-α treatment in IP-DPSCs did not. Therefore, regulation of telomerase activity in IP-DPSCs may accelerate their impaired dentin/pulp regeneration and immunosuppressive functions.

In conclusion, the present findings indicated that IFN-γ treatment improved the impaired IP-DPSC functions of dentin/pulp regeneration and immunosuppressive regulation. These effects were modified by telomerase activity, but independent of the NF-κB pathway. This functional gain study of disease-derived DPSCs may contribute to achieving not only autologous stem cell-approached pulp tissue engineering, but also to develop novel pulp-capping agents targeted to recipient diseased stem cells in future endodontic therapy (Supplementary Figure 7).

## Methods

### Ethics statement and human participants

All human samples were obtained as discarded biological/clinical samples from systemically healthy donors (19–25 years old) in Kyushu University Hospital. Extracted dental pulp tissue (n = 4) from permanent teeth that were diagnosed with irreversible pulpitis was aseptically collected under pulpectomy. Healthy pulp tissue (n = 3) was obtained from participants with healthy impacted third molars (19–23 years old). Human PBMNCs were provided by healthy volunteers (26–38 years old). The procedures for using human samples were conducted in accordance with the Declaration of Helsinki and approved by the Kyushu University Institutional Review Board for Human Genome/Gene Research (Protocol Number: 393-01). Written informed consent was obtained from all subjects. All experimental protocols were approved by the Institutional Animal Care and Use Committee of Kyushu University (Protocol Number: A21-044-1). The methods were carried out in accordance with the approved guidelines.

### Mice

BALB/cAJcl-nu/nu mice (female, 7–10 weeks old) were purchased from CLEA Japan (Tokyo, Japan) and used under an institutionally approved animal research protocol (Kyushu University protocol #A21-044-1).

### Histology of dental pulp tissue of human teeth diagnosed with irreversible pulpitis

Histological analysis with hematoxylin and eosin (H&E) staining and immunohistochemistry for human dental pulp tissue was performed as described in Supplementary Methods^49^. Mouse anti-STRO-1 IgM antibody (R&D Systems, Minneapolis, MN, USA) and non-immune mouse IgM (R&D Systems) were used for immunohistochemical analysis.

### Isolation and culture of DPSCs

Stem cells from the dental pulp tissue of human permanent teeth were isolated as described in Supplementary Methods^7^,^50^. Briefly, the dental pulp tissue was digested with 0.3% collagenase type I (Worthington Biochemicals, Lakewood, NJ, USA) and 0.4% dispase II (Sanko Junyaku, Tokyo, Japan) for 60 min at 37 °C. The single cells obtained were seeded. After 3 hours, the cultures were washed and cultured with a regular medium consisting of 15% fetal bovine serum (Equitech-Bio, Kerrville, TX, USA), 100 μM L-ascorbic acid 2-phosphate (Wako Pure Chemicals, Osaka, Japan), 2 mM L-glutamine (Nacalai Tesque, Kyoto, Japan), and antibiotics containing 100 U/ml penicillin and 100 μg/ml streptomycin (Nacalai Tesque) in Alpha Modification of Eagle’s Medium (Invitrogen, Waltham, MA, USA). After forming well-attached colonies, the cells were passed for expansion.

### CFU-F assay

The CFU-F assay was analyzed as described in Supplementary Methods^49^,^50^,^51^.

### Immunophenotype analysis

Cell surface antigens of IP-DPSCs and healthy DPSCs were assayed by flow cytometric analysis according to Supplementary Methods^49^,^50^,^51^. All primary antibodies are summarized in Supplementary Table 1.

### Population-doubling and BrdU incorporation assays

Population-doubling and BrdU incorporation assays were performed as described in Supplementary Methods^49^,^50^,^51^.

### Telomerase activity assay

Telomerase activity was measured by a telomere repeat amplification protocol (TRAP) assay using a quantitative telomerase detection kit (Allied Biotech, Ijamsville, MD, USA) with RT-qPCR as described in Supplementary Methods^49^,^50^,^51^.

### *In vitro* multidifferentiation capacity assay

Assays for multipotency into odontoblasts/osteoblasts, adipocytes, endothelial cells, and neural cells were performed as described in Supplementary Methods^49^,^50^.

### Assays for *in vivo* dentinogenic ability and self-renewal capacity

To analyze *in vivo* dentinogenic capacity, the cells were implanted subcutaneously with HA/TCP ceramic powders (40 mg, Zimmer Inc., Warsaw, IN, USA) into BALB/cAJcl-nu/nu mice as described in Supplementary Methods^49^,^50^,^51^,^52^ (Supplementary Figure 2). Eight weeks after the surgery, the implants were analyzed histologically. For self-renewal assay, the cells were sequentially transplanted as described in Supplementary Methods^49^,^50^ (Supplementary Figure 2).

### *In vivo* dentin regeneration on human dentin

*In vivo* dentin regeneration on human dentin was performed as described in Supplementary Methods^8^ (Supplementary Figure 6).

### Histological assay for implant tissue

Harvested implant tissue was treated for histochemical, immunohistochemical, and immunofluorescent assays as described in Supplementary Methods^49^,^50^,^51^. The newly formed mineralized tissue area in each field was measured and shown as a percentage of the total tissue area^44^,^52^.

### Single colony-derived cell assay

Single colony-derived cells were assayed by population-doubling, BrdU incorporation and *in vitro* dentinogenesis as described in Supplementary Methods^19^,^49^,^50^.

### Treatment with IFN-γ and TNF-α

IP-DPSCs were stimulated with IFN-γ (100 ng/ml; PeproTech, Rocky Hill, NJ, USA) or TNF-α (100 ng/ml; PeproTech) at 37 °C for 0, 30, 60, and 120 minutes before analyzing an expression of NF-κB and its phosphorylated NF-κB under a serum-depleted condition. IP-DPSCs were pretreated with either cytokine at 37 °C for 24 hours before the transplant experiments under a serum-added condition.

### Gene expression assay

Semi-quantitative RT-PCR and RT-qPCR assays were performed as described in Supplementary Methods^53^,^54^. The specific primer pairs and TaqMan probes (Applied Biosystems, Foster City, CA, USA) are summarized in Supplementary Tables 2 and 3.

### PBMNC viability assay

Cell viability of PBMNCs under co-culture with IP-DPSCs or healthy DPSCs was performed as described in Supplementary Methods^9^,^24^ (Supplementary Figure 5a). Some PBMNCs were stimulated with ConA (10 μg/ml; Sigma-Aldrich, St. Louis, MO, USA). IP-DPSCs and healthy DPSCs were also pretreated with inhibitors for COX, NOS, IDO, indomethacin (20 μM; Sigma-Aldrich), L-NAME (1 mM; Sigma-Aldrich), and 1-MT (500 μM; Sigma-Aldrich), as well as antibodies neutralized to human IL-10 (10 μg/ml; R&D Systems), human TGF-β1 (10 μg/ml; R&D Systems), or an isotype-matched monoclonal antibody (10 μg/ml; R&D Systems). The collected CM was used for L-kynurenine and IL-10 measurements.

### IDO activity assay

IDO activity was analyzed by measuring L-kynurenine in CMs of IP-DPSCs or healthy DPSCs co-cultured with ConA-activated PBMNCs according to a previous study^9^.

### IL-10 measurement

Human IL-10 in culture supernatant was analyzed by ELISA in fresh co-culture CMs of IP-DPSCs or healthy DPSCs with ConA-activated PBMNCs using the Human IL-10 Quantikine ELISA kit (R&D Systems) according to the manufacturer’s instructions.

### Apoptosis assay

T cell-mediated apoptosis assay in IP-DPSCs and healthy DPSCs was performed as described in Supplementary Methods^25^,^26^,^51^ (Supplementary Figure 5b). IP-DPSC and healthy DPSCs was also treated with anti-FasL antibody (1 μg/mL; eBioscience, San Diego, CA, USA). Apoptosis was assayed by toluidine blue staining and the TUNEL method.

### Western blot analysis

Western blot analysis was performed as described in Supplementary Methods^51^,^52^. The primary antibodies used in this assay were NF-κB p65 (Cell Signaling Technology, Danvers, MA, USA), phosphorylated NF-κB p65 (Cell Signaling Technology), and β-actin (Sigma-Aldrich).

### Statistical analysis

All data are expressed as the mean ± s.e.m. of, at least, triplicate determinations. Comparisons between two groups were analyzed by independent two-tailed Student’s t-tests. Multi-group comparisons were analyzed by one-way repeated measures analysis of variance followed by Tukey’s post hoc test. P-values less than 0.05 were considered statistically significant.

## Additional Information

**How to cite this article**: Sonoda, S. *et al*. Interferon-gamma improves impaired dentinogenic and immunosuppressive functions of irreversible pulpitis-derived human dental pulp stem cells. *Sci. Rep*. **6**, 19286; doi: 10.1038/srep19286 (2016).

## Supplementary Material

Supplementary Information
