# Supplementary material for: Interferon-gamma improves impaired dentinogenic and immunosuppressive functions of irreversible pulpitis-derived human dental pulp stem cells
Source: Sci Rep. 2016 Jan 18;6:19286. doi: 10.1038/srep19286 (PMC4726054; doi:10.1038/srep19286)
Supplement: Supplementary Information [file srep19286-s1.pdf]

Supplementary Information

Interferon-gamma improves impaired dentinogenic and immunosuppressive functions of irreversible pulpitis-derived human dental pulp stem cells

Soichiro Sonoda[1,2], Haruyoshi Yamaza[3], Lan Ma[1,3,4], Yosuke Tanaka[1], Erika Tomoda[1,3], Reona Aijima[1,5], Kazuaki Nonaka[3], Toshio Kukita[1], Songtao Shi[5], Fusanori Nishimura[2], Takayoshi Yamaza[1]*

Departments of [1]Molecular Cell Biology and Oral Anatomy, [2]Periodontology and [3]Pediatric Dentistry, Kyushu University Graduate School of Dental Science, Fukuoka, Japan

[4]Department of Pediatric Dentistry, Guanghua School of Stomatology, Hospital of Stomatology, Sun Yat-sen University, Guangzhou, China

[5]Department of Histology and Neuroanatomy, Faculty of Medicine, Saga University, Saga, Japan

[6]Department of Anatomy and Cell Biology, School of Dental Medicine, University of Pennsylvania, PA, USA

*Correspondence: Takayoshi Yamaza, DDS, PhD
Department of Molecular Cell Biology and Oral Anatomy
Kyushu University Graduate School of Dental Science
3-1-1 Maidashi, Higashi-ku, Fukuoka 812-8582, Japan
Tel: +81-92-642-6305
Fax: +81-92-642-6304
E-mail: yamazata@dent.kyushu-u.ac.jp

Supplementary Methods

Isolation and culture of dental pulp stem cells

Dental pulp tissues were digested with 0.3% collagenase type I (Worthington Biochemicals, Lakewood, NJ) and 0.4% dispase II (Sanko Junyaku, Tokyo, Japan) for 60 min at 37°C, and were passed through a 70-μm filter to obtain single cell suspension. Single mononuclear cells were seeded at 0.1x10^{6} per flask on T-75 flasks. After three hours, the cultures were washed with sterilized phosphate-buffered saline (PBS). The adherent cells were incubated with a regular medium consisting of 15% fetal bovine serum (Equitech-Bio, Kerrville, TX), 100 μM L-ascorbic acid 2-phosphate (Wako Pure Chemicals, Osaka, Japan), 2 mM L-glutamine (Nacalai Tesque, Kyoto, Japan), and antibiotics containing 100 U/ml penicillin and 100 μg/ml streptomycin (Nacalai Tesque) in alpha Modification of Eagle's Medium (αMEM) (Invitrogen, Waltham, MA). The regular medium was changed twice a week throughout the culture. After growing attached colonies well, the cells were passed and maintained in the regular medium. Generally, passaged 3 cells (P3) were used for further experiments.

Histology of dental pulp tissues of human teeth diagnosed as an irreversible pulpitis

Human dental pulp tissues were extracted from permanent teeth, that were clinically diagnosed as an irreversible pulpitis, and were fixed with 4% paraformaldehyde (PFA) in PBS and immersed in O.C.T. compound (Sakura Finetek Japan, Tokyo, Japan). The frozen specimens were cut into 6-μm thick sections. Some sections were stained with hematoxylin and eosin (H&E). The others were immunostained with mouse anti-STRO-1 IgM antibody using SuperPicture kit (Invitrogen). Non-immune mouse IgM were used for negative controls for immunohistochemistry. The sections were observed under an Axio Imager M2 (Zeiss, Oberkochen, Germany).

Colony forming unit-fibroblasts (CFU-F) assay

Isolated mononuclear cells were seeded at 1 x10^5 per dish on 100-mm culture dishes with the regular medium. The cultures were washed with PBS, and adherent cells were cultured for 16 days. The cultures were treated with a mixture of 0.1% toluidine blue and 4% paraformaldehyde (PFA) in PBS, pH 74, overnight. Cell clusters containing >50 cells were counted as single colonies (CFU-F) under a Primovert microscope

microscope (Zeiss). The CFU-F numbers were measured in three independent assays *per* each tooth.

Immunophenotype analysis

P3 IP-DPSCs and healthy DPSCs (each $1x10^6$/100 μl in PBS containing 5% heat-inactivated FBS [Equitech-Bio]) were stained for 45 minutes on ice with fluorescent-conjugated specific antibodies (1 μg/100 μl) against cell surface markers. Corresponding isotype-matched antibodies (1 μg/100 μl) were used for negative controls. All primary antibodies used in this assay were listed on **Supplementary Table 1**. The samples were then washed with PBS containing 5% heat-inactivated FBS (Equitech-Bio). Total $10x10^3$ cells were measured on a FACSVerse flow cytometer (BD Biosciences, San Jose, CA) and analyzed by using BD FACSuit software (BD Biosciences). The number (percentage) of positive cells was determined in comparison with the corresponding control cells in which a false-positive rate of less than 1% was accepted.

Population doubling (PD) assay

Cells were seeded on T-75 culture flasks with the regular medium. When the cells reached at a sub-confluent condition, the cells were passed and sub-cultured. These steps were repeated until the cells lost their dividing ability. The PD score was calculated at every passage according to the equation: $\log_2$ (number of final harvested cells/number of initial seeded cells). The total score determined the PD scores. The PD scores were calculated in three independent assays per each dental pulp sample.

Bromodeoxyuridine (BrdU) incorporation assay

P3 IP-DPSCs and healthy DPSCs were seeded at $1\text{x}10^3$ per well on 8-well chamber slides (Nunc, Waltham, MA), and were cultured in the growth medium. The cells were treated with BrdU reagent (1:100) (Invitrogen) for 24 hours, and stained by using a BrdU staining kit (Invitrogen). The samples were then lightly stained with hematoxylin. Seven areas per each well were randomly selected, and captured with an Axio Imager M2 microscope (Zeiss). BrdU-positive and BrdU-negative nuclei numbers were calculated in each image by an Image-J software (National Institutes of Health [NIH],

Bethesda, MD). Cell proliferation capacity was shown as a percentage of BrdU-positive nuclei over total nucleated cells. The BrdU positive rate was calculated in three independent assays per each sample.

Telomerase activity assay

Telomerase activity was measured by a telomere repeat amplification protocol (TRAP) assay using a quantitative telomerase detection kit (Allied Biotech, Ijamsville, MD) applied with a Light Cycler 96 real-time PCR system (Roche, Basel, Switzerland). P3 IP-DPSCs and healthy DPSCs were collected for telomerase activity test. HEK293T cells were used for positive control. Some samples from each cell group were heated at 85°C for 10 min and used as negative control tests. The average starting quantity (SQ) of fluorescence units was used to compare the telomerase activity among the samples. Telomerase activity was calculated in three independent assays per each sample.

In vitro multidifferentiation capacity assay

P3 cells were assays for multidifferentiation capacity into odontoblasts/osteoblast, adipocytes, endothelial cells, and neural cells Each assay/test was repeated in three independent assays per each sample.

In vitro dentinogenic/osteogenic induction assay: P3 cells were seeded at $5x10^3$ per dish on 60-mm culture dishes, and cultured in the regular medium until they reached at a confluent condition. The medium was then changed to a dentinogenic/osteogenic induction medium. The dentinogenic/osteogenic induction medium consisted of 15% FBS (Equitech-Bio), 2 mM L-glutamine (Nacalai Tesque), 100 μM L-ascorbic acid 2-phosphate (WAKO Pure Chemical), 1.8 mM potassium dihydrogen phosphate (Sigma-Aldrich, St. Louis, MO), 10 nM dexamethasone (Sigma-Aldrich) and antibiotics containing 100 U/ml penicillin and 100 μg/ml streptomycin (Nacalai Tesque) in αMEM (Invitrogen). The dentinogenic/osteogenic medium was changed twice a week. The cultures were harvested one week after the induction, and were analyzed for dentinogenic/osteogenic specific gene assay by semi-quantitative reverse transcription-polymerase chain reaction (RT-PCR) and quantitative RT-PCR (qRT-PCR). Independent control mRNAs were normalized to 1. They also used for

alkaline phosphatase (ALP) activity test with LabAssay ALP (WAKO Pure Chemical) according to the manufacture's instruction. For calcium accumulation assay, the cultures were stained with 1% Alizarin Red-S (Sigma-Aldrich) four weeks after the induction. The mineralized area was measured by Image J (NIH) and shown as a percentage of Alizarin Red-positive area over the total area.

In vitro adipogenic induction assay: Cells (P3, 5×10^3/dish) were cultured at a confluent condition with the regular medium, and were then induced in an adipogenic medium with the growth medium supplemented with 500 μM isobutyl-methylxanthine (Sigma-Aldrich), 60 μM indomethacin (Sigma-Aldrich), 0.5 μM hydrocortisone (Sigma-Aldrich) and 10 μM insulin (Sigma-Aldrich). Six weeks after the induction, the cultures were stained with 0.3% Oil red O (Sigma-Aldrich) to detect lipid droplets, and observed under a Primovert microscope (Zeiss). Oil red O was extracted with isopropanol from the samples, and the absorbance of the extracts were then measured at 520 nm with a Multiskan GO spectrophotometer (Thermo Scientific, Waltham, MA). Adipocyte-specific genes were also analyzed by semi-quantitative RT-PCR and qRT-PCR.

In vitro endothelial cell induction assay: P3 Cells were seeded at $1x10^3$ cells per well on fibronectin-coated 8-well chamber slides (Nunc) and cultivated with endothelial growth medium 2 kit (Lonza, Basel, Switzerland) for 7 days. The medium was changed every 2 days. The cells were assayed by immunofluorescence with anti-CD31 antibody (Affymetrix, San Diego, CA) and Alexa fluor 647-conjugated secondary antibody (Dako, Glostrup, Denmark). The nuclei were stained by 4',6-diamidino-2-phenylindole (DAPI) (Dojindo Laboratories, Kumamoto, Japan). Seven areas per each well were randomly selected, and captured under an Axio Imager M2 microscope (Zeiss). CD31-positive and CD31-negative cell numbers were calculated in each image by an Image-J software (NIH). Endothelial cell differentiation rate was shown as a percentage of CD31-positive cells over total cells. The CD31 positive rate was calculated in three independent assays *per* each sample.

Neuronal differentiation: Cells were plated at $1x10^4$ cells per well in poly-D-lysine/laminin-coated 8-well chamber slides (Nunc), and were cultured in Neurobasal A (Invitrogen) supplemented with 1xN2 supplement (Invitrogen), 10 ng/ml fibroblast growth factor 2 (PeproTech, Rocky Hill, NJ), and 10 ng/ml epidermal growth

factor (PeproTech), and antibiotics containing 100 U/ml penicillin and 100 μg/ml streptomycin (Nacalai Tesque), and cultured for 21 days. The medium was changed with 50% of fresh medium every 3–4 days throughout the experimental period. The cultures were assayed by immunofluorescence with antibodies against glial fibrillary acidic protein (GFAP) (Sigma-Aldrich), neurofilament M (Sigma-Aldrich), or tubulin βIII (Sigma-Aldrich). The samples were incubated with and Alexa fluor 647-conjugated secondary antibody (DAKO), and stained by DAPI (Dojindo Laboratories), and observed under Axio Imager M2 microscope (Zeiss). Seven areas per each well were randomly selected, and captured under an Axio Imager M2 microscope (Zeiss). GFAP-, neurofilament M-, and tubulin βIII-positive and cell numbers were calculated in each image by an Image-J software (NIH). Neural cell differentiation rate was shown as a percentage of GFAP-, neurofilament M-, and tubulin βIII-positive cells over total cells. The GFAP-, neurofilament M-, and tubulin βIII-positive rate was calculated in three independent assays per each sample.

Controls for multidifferentiation: DPSCs or IP-DPSCs were cultured in the growth medium as indicated periods and used for independent marker expression assay.

Assays for in vivo dentinogenic ability and self-renewal capacity

To analyze in vivo dentinogenic capacity, P3 cells ($4.0x10^6$) cultured under the regular condition were mixed with hydroxyapatite/tricalcium phosphate (HA/TCP) ceramic powders (40 mg, Zimmer Inc., Warsaw, IN) (**Supplementary Figure 2**). The mixture was implanted subcutaneously into the dosal surface of 8-10-week-old Balb/cAJcl-*nu/nu* immunocompromised mice. Eight weeks after the surgery, the implants were harvested, and treated for histological analysis.

To analyze *in vivo* self-renewal capacity, cells were sequentially transplanted (**Supplementary Figure 2**). P3 cells ($4x10^6$) were primary implanted with HA/TCP carrier (40 mg) (Zimmer) into Balb/c *nu*/*nu* mice. Eight weeks after the transplantation, the primary implants were harvested and treated with 0.4% dispase II (Sanko Junyaku) for 60 min at 37°C. Obtain cells from the implants were seeded and cultured until they formed attached CFU-F. CFU-F-forming cells ($4x10^6$) were secondary transplanted with HA/TCP carriers (40 mg) (Zimmer) under dorsal skin of immunocompromised mice for eight weeks. Secondary implants were also assayed histologically.

In vivo dentin regeneration on human dentin

Inner surface of root canals of human molars was scraped thinly, and were treated with 1% acetic acid for 10 min at room temperature followed by washed with PBS (**Supplementary Figure 6**). The root foramens were then sealed with an absorbable gelatin sponge Spongel (Astellas Pharma, Tokyo, Japan). Cell suspension ($2.0x10^6$) in the regular medium was incubated in the root canal space at 37°C for 24 hours. The culture medium was then removed. IP-DPSC-loaded tooth roots were subcutaneously implanted under the dorsal skin of Balb/c *nu*/*nu* mice. Eight weeks after the surgery, the implants were harvested, and treated for histological analysis.

Histological assay for implant tissues

Harvested implant tissues were fixed with 4% PFA in PBS overnight at 4^oC, and were decalcified with 10% EDTA solution (pH 8.0). The samples were dehydrated and embedded in paraffin, and were cut into 6-μm-thick sections. Paraffin sections were treated with hematoxylin and eosin (H&E) staining or Alinine blue staining. H&E

stained images were treated with invert color process by Photoshop software (Adobe Systems, San Jose, CA). For immunohistochemistry, paraffin sections were incubated with anti-human mitochondria antibody (Millipore, Billerica, MA), and treated with using SuperPicture kit (Invitrogen) according to the manufacture's instruction. The sections were then stained with hematoxylin. For immunofluorescence, paraffin sections were treated with anti-human CD146 antibody (eBioscience) or anti-dentin phosphoprotein (DSPP) (Santa Cruz Biotechnology, Santa Cruz, CA), and then incubated with Alexa fluor 647-conjugated secondary antibody (DAKO). They were finally stained with DAPI (Dojindo Laboratories). All of the sections were observed under an Axio Imager M2 microscope (Zeiss). To analyze *in vivo* regenerative capacity, seven fields were randomly selected from H&E stained sections. Newly formed mineralized tissue area in each field was measured by Image-J software (NIH), and the results were shown as a percentage of mineralized tissue area over total tissue area.

Single colonies-derived cell assay

Cells isolated from pulp tissues were seeded at 1, 2 or 4 cells per well on 24-well multiplates with the regular medium. Wells contained more than two attached cells were excluded from further culture. Only single cell-attached wells were cultured for 14-16 days. The single colony-forming cells were used for further assays of population doubling, BrdU incorporation and *in vitro* dentinogenesis.

Gene expression assay

Total RNAs were extracted from cultured cells with TRIzol (Invitrogen), were digested with DNase I (Promega, Madison, WI), and were purified using an RNeasy Mini kit (Qiagen, Venlo, Netherlands). One microgram of purified RNA was reverse-transcribed with Revertra Ace qPCR kit (TOYOBO, Osaka, Japan). For semi-quantitative RT-PCR analysis, cDNA was amplified with a T-100 thermal cycler (Bio-Rad, Hercules, CA) using Quick Taq HS DyeMix (TOYOBO) and specific primer pairs (**Supplementary Table 2**). Five µl of each amplified PCR product was analyzed by 2 % agarose gel electrophoresis, and visualized by ethidium bromide staining. For qRT-PCR assay, cDNA was subsequently amplified using a TaqMan Gene Expression Master Mix

(Applied Biosystems, Foster City, CA) and target TaqMan probes (Applied Biosystems) (**Supplementary Table 3**) with a Light Cycler 96 real-time PCR system (Roche). 18S ribosomal RNA was used for normalization.

PBMNC cell viability assay

PBMNCs were separated from whole peripheral blood using Ficoll-Paque (GE Healthcare Life Sciences, Pittsburgh, PA). P3 IP-DPSCs and healthy DPSCs were γ-irradiated at a dose of 30 Gy with an MBR-1520R-3 (Hitachi, Tokyo, Japan). γ-irradiated IP-DPSCs and healthy DPSCs were plated at a number of 0, 1×10^3, 10×10^3, 100×10^3 per well on 96-well multiplates, and were incubated overnight. Human PBMNCs were then directly loaded at 100×10^3 cells per well over pre-plated γ-irradiated IP-DPSCs and healthy DPSCs in the presence or absence of concanavarin A (ConA) (10 μg/ml; Sigma-Aldrich) under a complete medium. PBMNCs were also indirectly co-cultured with IP-DPSCs or healthy DPSCs using a transwell system. Gamma-irradiated IP-DPSCs or healthy DPSCs were seeded on a lower well, and PBMNCs were loaded on the upper wells. Some γ-irradiated IP-DPSCs and healthy

DPSCs alone and ConA-stimulated and ConA-free PBMNCs alone were also cultured. The complete medium was consisted of RPMI-1640 medium (Sigma-Aldrich) with 10% heat-inactivated FBS (Equitech-Bio), 2 mM L-glutamine (Nacalai Tesque), 1 mM sodium pyruvate (Nacalai Tesque), and antibiotics containing 100 U/ml penicillin and 100 μg/ml streptomycin (Nacalai Tesque) (**Supplementary Figure 3a**). PBMNCs and IP-DPSCs/healthy DPSCs were cultured in a transwell system by using HTS Transwell-96permeable support (Corning, Acton, MA). IP-DPSCs and healthy DPSCs were seeded on a lower chamber well of PBMNCs were seeded in an upper chamber (**Supplementary Figure 3b**). After 72 hours, cell viability in the floating cells was assayed by using Cell Counting Kit-8 (Dojindo Laboratories) according to the manufactures' instructions, and was then measured at 450 nm with a Multiskan GO spectrophotometer (Thermo Scientific). Some IP-DPSCs or healthy DPSCs were pretreated with inhibitors for cyclooxygenase (COX), nitric oxide synthase (NOS), and indoleamine 2,3-dioxygenase (IDO), indomethacin (20 μM; Sigma-Aldrich), *N*-nitro-L-arginine methyl ester (L-NAME) (1 mM; Sigma-Aldrich), 1-methyl-L-tryptophan (1-MT) (500 μM; Sigma-Aldrich), respectively, and neutralized

antibodies to human IL-10 (10 μg/ml; R&D Systems), human TGF-β1 (10 μg/ml; R&D Systems), or an isotype-matched monoclonal antibody (10 μg/ml; R&D Systems) before PBMNCs were loaded. The viability was measured in three independent assays per each sample (**Supplementary Figure 5a**). Conditioned medium (CM) were also collected, and were centrifuged at 15,000 rpm for 30 min. They were filtrated with a 0.45-μm pore sized filter, and were applied to L-kynurenine assay and IL-10 measurement.

Apoptosis assay

PBMNCs were loaded at $1x10^6$ per well plate-bounded with anti-human CD3 antibody (1 μg/ml; eBioscience) in the complete medium supplemented with soluble anti-human CD28 antibody (1 μg/ml; eBioscience) onto 24-well multiplates for three days (**Supplementary Figure 5b**). IP-DPSCs or healthy DPSCs were plated at $200x10^3$ per well onto another 24-well multiplates, and were incubated overnight. Activated PBMNCs ($1x10^6$) were loaded directly on IP-DPSCs or healthy DPSCs, and were co-cultured in the complete medium in the absence or presence of anti-FasL antibody (1

μg/ml; MBL, Nagoya, Japan). Three days after the coculture, the cultured wells were washed with PBS, and were stained with 2% PFA and 2% toluidine blue. One day after the co-culture, the wells washed with PBS, and were stained for cell death by TdT-mediated dUTP nick end labeling (TUNEL) method using ApoTag Peroxidase In Situ Apoptosis Detection kit (Millipore) according to the manufacture's instructions. Seven fields per each well were randomly selected, and captured with an Axio Imager M2 microscope (Zeiss). TUNEL-positive and TUNEL-negative nuclei numbers were calculated in each image by an Image-J software (National Institutes of Health [NIH], Bethesda, MD). Cell death rate was shown as a percentage of TUNEL-positive nuclei over total nucleated cells. The TUNEL positive rate was calculated in three independent assays per each sample.

Western blot analysis

Cultured cells were collected at the corresponding period. They were lysed in M-PER mammalian protein extraction reagent (Life Technologies) supplemented with proteinase inhibitor cocktail (Nacalai Tesque) and PhoSTOP phosphatase inhibitor

(Roche), and total proteins were then extracted. The protein samples were mixed with NuPage LDS Sample buffer (Life Technologies), and were treated at 70°C for 7 min. Ten μg of protein were separated by 10% TGX gels (Bio-Rad), and transferred to a polyvinylidene difluoride membrane using Trans-Blot Turbo Transfer System RTA Transfer Kit (Bio-Rad) with a Trans-Blot Turbo blotting system (Bio-Rad) according to the manufacture's instrument. The membranes were blocked with 5% skimmed milk in Tris-buffered saline (150 mM NaCl and 20 mM Tris–HCl, pH 7.2) for one hour at room temperature, and then incubated with primary antibodies overnight at 4°C. The primary antibodies used in this assay were against to NF-κB p65 (Cell Signaling Technology, Danvers, MA), phosphorylated NF-κB p65 (Cell Signaling Technology), Fas (Santa Cruz Biotechnology) and β-actin (Sigma-Aldrich). They were treated with horseradish peroxidase-conjugated donkey anti-rabbit or anti-mouse IgG secondary antibody (1:1000; Santa Cruz Biotechnology) for one hour at room temperature. The membranes were visualized using SuperSignal West Pico (Thermo Scientific), and captured under an ImageQuant LAS 4010 (GE Healthcare Life Sciences, Pittsburgh, PA).

Supplementary Tables

Supplementary Table 1: The list of primary antibodies for flow cytometry.

Names of antibodies	Types of antibodies	Names of Suppliers
anti-CD14 antibody	R-PE -conjugated	eBioscience (San Diego, CA)
anti-CD34 antibody	R-PE-conjugated	eBioscience (San Diego, CA)
anti-CD45 antibody	R-PE-conjugated	eBioscience (San Diego, CA)
anti-CD73 antibody	R-PE-conjugated	eBioscience (San Diego, CA)
anti-CD90 antibody	R-PE-conjugated	eBioscience (San Diego, CA)
anti-CD105 antibody	R-PE-conjugated	eBioscience (San Diego, CA)
anti-CD146 antibody	R-PE-conjugated	eBioscience (San Diego, CA)
anti-STRO-1 antibody	R-PE-conjugated	abcam (Cambridge, MA)

R-PE: R-phycoerythrin

Supplementary Table 2: The list of primer pairs for RT-PCR.

alkaline phosphatase (*ALP*) (X14390)

sense: 5’-ACGTGGCTAAGAATGTCATC-3’ (nucleotide 322-341)

antisense: 5’-CTGGTAGGCGATGTCCTTA-3’ (nucleotide 779-797)

sense: 5’-ACGTGGCTAAGAATGTCATC-3’ (nucleotide 322-341)

CD271 (NM_002507)

sense: 5’-CACCTCCAGAACAAGACCTC-3’ (775-794)

antisense: 5’-GAGCCGTTGAGAAGCTTCTC-3’ (1167-1186)

dentin sialophosphoprotein (*DSPP*) (NM_014208)

sense: 5′-GGCAGTGACTCAAAAGGAGC-3′ (1630-1649)

antisense: 5′-TGCTGTCACTGTCACTGCTG-3′ (1815-1834)

glyceraldehyde 3-phosphate dehydrogenase (*GAPDH*) (M33197)

sense: 5′-AGCCGCATCTTCTTTTGCGTC-3′ (12-32)

antisense: 5′-TCATATTTGGCAGGTTTTTCT-3′ (807-827)

lipoprotein lipase (*LPL*) (X14390)

sense: 5′-ATGGAGAGCAAAGCCCTGCTC-3′ (118-138)

antisense: 5′-GTTAGGTCCAGCTGGATCGAG-3′ (661-681)

NANOG (NM_024865)

sense: 5’-TCCTCCATGGATCTGCTTATTCA-3’ (382-404)

antisense: 5’-CAGGTCTTCACCTGTTTGTAGCTGAG-3’ (616-641)

NESTIN (NM_006617)

sense: 5’-CAGCGTTGGAACAGAGGTTGG-3’ (852-872)

antisense: 5’-TGGCACAGGTGTCTCAAGGGTAG-3’ (1218-1240)

NOTCH1 (NM_017617)

sense: 5’-CACCCAGAACTGCGTGCA-3’ (3840-3857)

antisense: 5’-GGCAGTCAAAGCCGTCGA-3’ (4547-4564)

octamer4 (OCT4) (NM_203289)

sense: 5’-GACAGGGGGAGGGGAGGAGCTAGG-3’ (1495-1518)

antisense: 5’-CTTCCCTCCAACCAGTTGCCCCAAAC-3’ (1613-1638)

osteocalcin (*OCN*) (X53698)

sense: 5′-CATGAGAGCCCTCACA-3′ (18-33)

antisense: 5′-AGAGCGACACCCTAGAC-3′ (316-332)

peroxisome proliferator activated receptor-γ2 (*PPARγ2*) (AB451337)

sense: 5′-CTCCTATTGACCCAGAAAGC-3′ (23-42)

antisense: 5′-GTAGAGCTGAGTCTTCTCAG-3′ (350-369)

runt-related gene 2 (RUNX2) (L40992)

sense: 5′-CAGTTCCCAAGCATTTCATCC-3 (880–900)

antisense: 5′-TCAATATGGTCGCCAAACAG-3′ (1304–1323)

Supplementary Table 3: The TaqMan primers and probes for qRT-PCR.

Gene names	Gene assay ID numbers
alkaline phosphatase (ALP)	Hs00171172_m1
dentin sialophosphoprotein (DSPP)	Hs00171962_m1
indoleamine 2,3-dioxygenase (IDO)	Hs00984148_m1
lipoprotein lipase (LPL)	Hs00173425_m1
osteocalcin (OCN)	Hs01587814_g1
peroxisome proliferator activated receptor-γ2 (PPARγ2)	Hs00234592_m1
ribosomal RNA, 18S	Hs03928985_g1
runt-related gene 2 (RUNX2)	Hs01047973_m1

Supplementary Figures

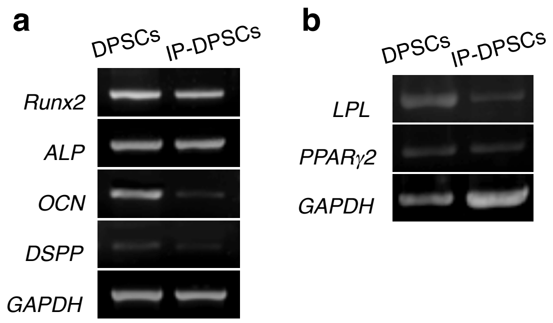

Supplementary Figure 1: Dentinogenic and adipogenic differentiation of IP-DPSCs.
(**a**) *In vitro* dentinogenic capacity. Semi-quantitative RT-PCR for odontoblast-specific genes including runt-related gene 2 (Runx2), ALP; alkaline phosphatase, osteocalcin (OCN), dentin sialophosphoprotein (DSPP). glyceraldehyde 3-phosphate dehydrogenase (GAPDH) is an internal control gene. (**b**) *In vitro* adipogenic capacity. Semi-quantitative RT-PCR for adipocyte-specific genes including lipoprotein lipase (LPL), peroxisome proliferator activated receptor-γ 2 (PPARγ 2). **a, b**: n=3 per group.

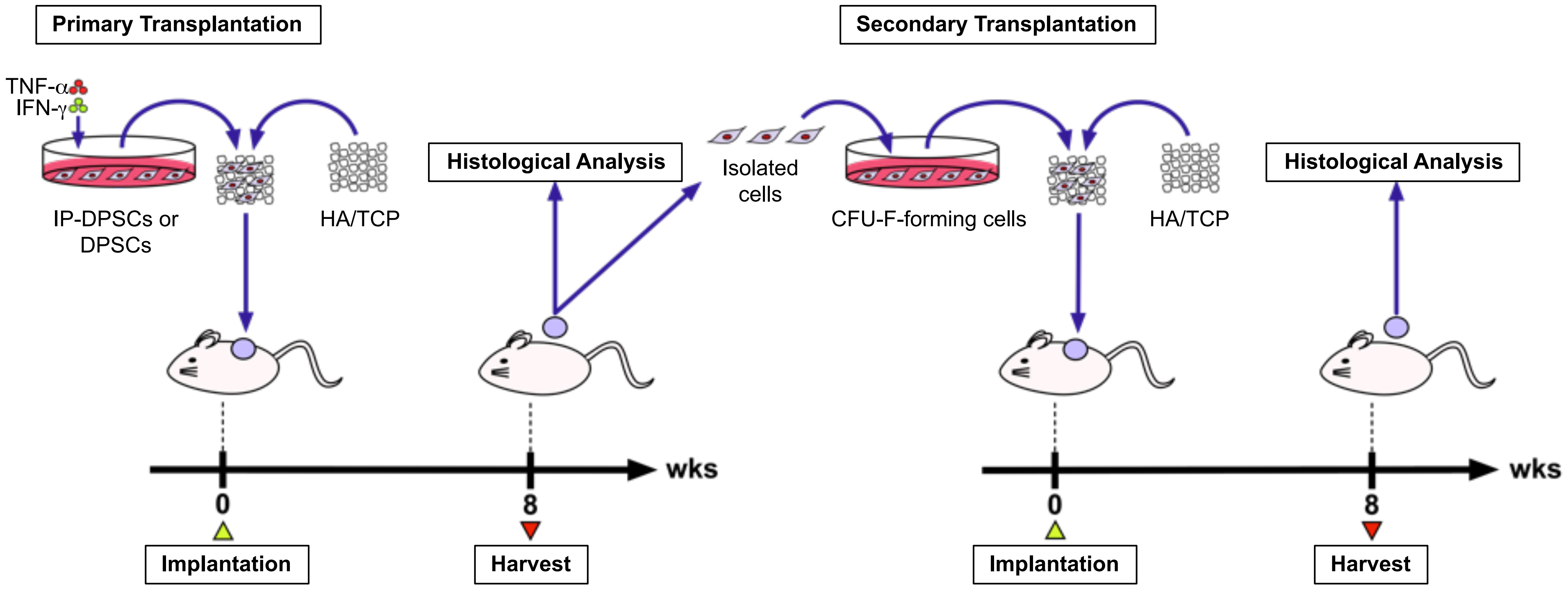

Supplementary Figure 2: A scheme of *in vivo* tissue regeneration and self-renewal assays of IP-DPSCs. IP-DPSCs were pre-treated with or without TNF-α (100 ng/ml) or IFN-γ (100 ng/ml) at 37°C for 24 hours before the transplantation. IP-DPSCs ($4.0x10^6$) or healthy DPSCs ($4.0x10^6$) were then transplanted with hydroxyl apatite/tricalcium phosphate (HA/TCP) carriers (40 mg) under the dorsal skin of immunocompromised mice. Eight weeks after the implantation, the primary transplants were harvested. Some transplants were used for histological analysis. Cells were isolated from the other primary transplants, and were seeded at low density to obtain attached colony-forming cells, colony forming unit-fibroblasts (CFU-F). The CFU-F cells were transplanted secondary into immunocompromised mice. The secondary transplants were harvested eight weeks after the implantation and analyzed morphologically.

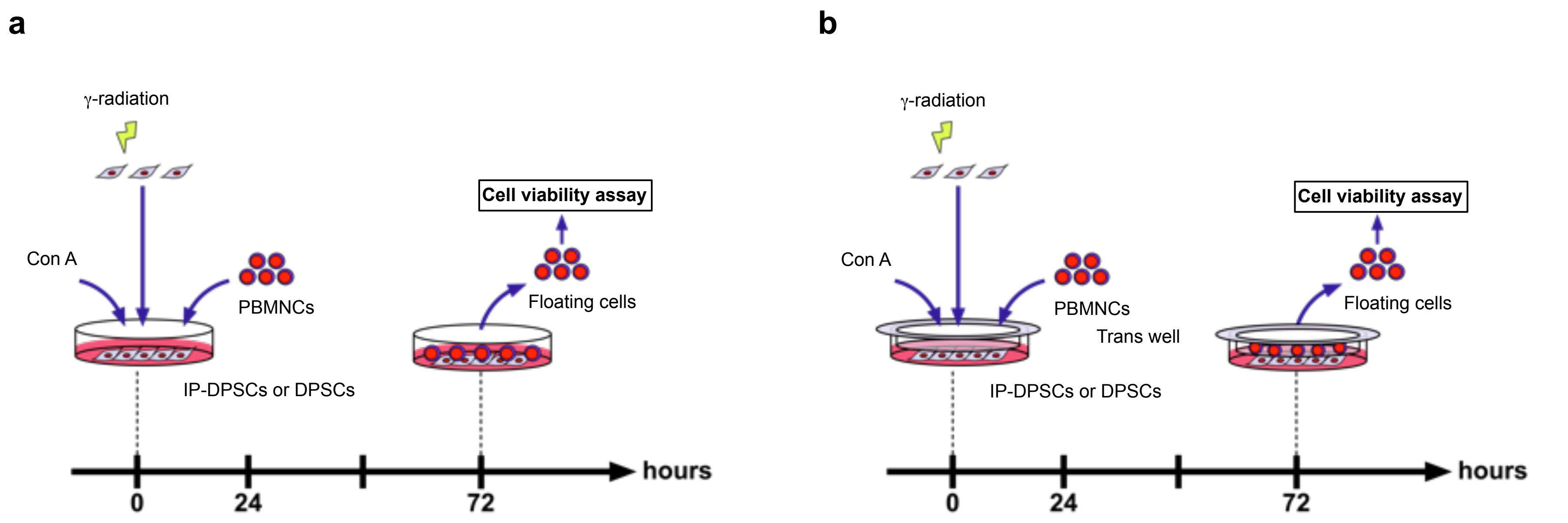

Supplementary Figure 3: Schemes of coculture systems of IP-DPSCs with PBMNCs. Effects of direct or indirect contact with IP-DPSCs on PBMNCs. (**a**) Gamma-irradiated IP-DPSCs or healthy DPSCs were seeded on a well. PBMNCs were loaded onto IP-DPSC cultures in the presence or absence of concanavalin A (ConA). After 72 hours, floating cells were collected, and were analyzed the cell viability. (**b**) Gamma-irradiated IP-DPSCs or healthy DPSCs were seeded on a lower well. PBMNCs were loaded onto the upper well by using transwell system in the presence or absence of concanavalin A (ConA). After 72 hours, both floating cells were collected, and were analyzed the cell viability.

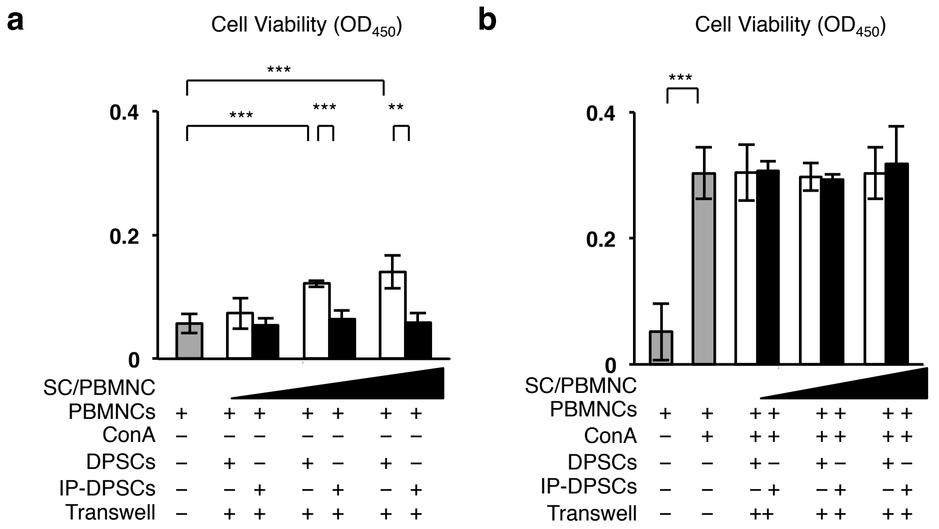

Supplementary Figure 4: Immunomodulatory effects of IP-DPSCs.

(**a, b**) Cell viability of human PBMNCs cultured with different ratios of DPSCs (SC) (SC/PBMNC = 0.01, 0.1, and 1) in the absence (**a**) or presence (**b**) of ConA under a transwell culture system. **A, b**, I: n=3 per group. $^{**}P<0.01$ and $^{***}P<0.005$. Graph bars show the means ± s.e.m.

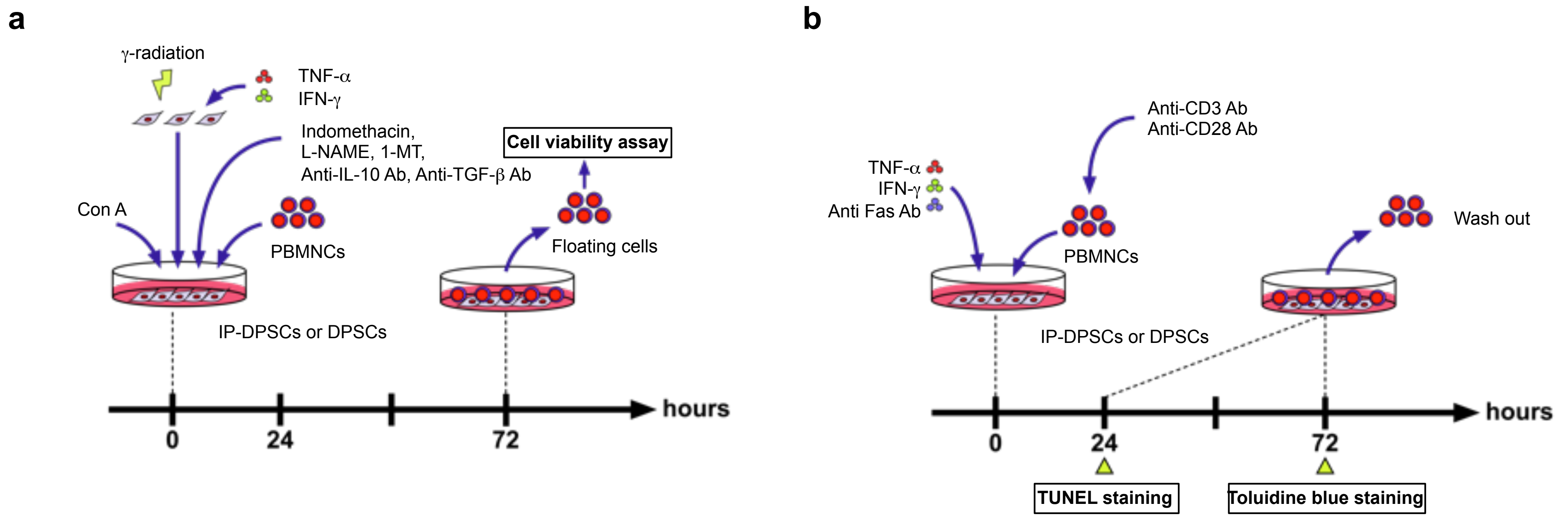

Supplementary Figure 5: Schemes of co-culture systems of IP-DPSCs with PBMNCs. Effects of TNF-α and IFN-γ pretreatment on PBMNCs co-cultured with IP-DPSCs. (**a**) Gamma-irradiated IP-DPSCs or healthy DPSCs were seeded with or without TNF-α (100 ng/ml) or IFN-γ (100 ng/ml). PBMNCs were loaded onto IP-DPSC cultures in the presence or absence of concanavalin A (ConA). The co-cultured treated with or without indomethacin, *N*-nitro-L-arginine methyl estel (L-NAME), 1-methyl-L-tryptophan (1-MT), anti-IL-10 antibody (Anti-IL10 Ab), and anti-TGF-β antibody (Anti-TGF-β Ab). After 72 hours, floating cells were collected, and were analyzed the cell viability. (**b**) PBMNCs were activated with plate-bounded anti-CD3 antibody (Anti-CD3 Ab) and soluble anti-CD28 antibody (Anti-CD28 Ab) for 3 days. IP-DPSCs or healthy DPSCs incubated with or without TNF-α (100 ng/ml) or IFN-γ (100 ng/ml) were co-cultured with the activated PBMNCs in the presence or absence of anti-Fas antibody (anti-Fas Ab). IP-DPSCs or healthy DPSCs were stained with 1% toluidine blue three days after the incubation, or were treated with TUNEL staining one day after the incubation.

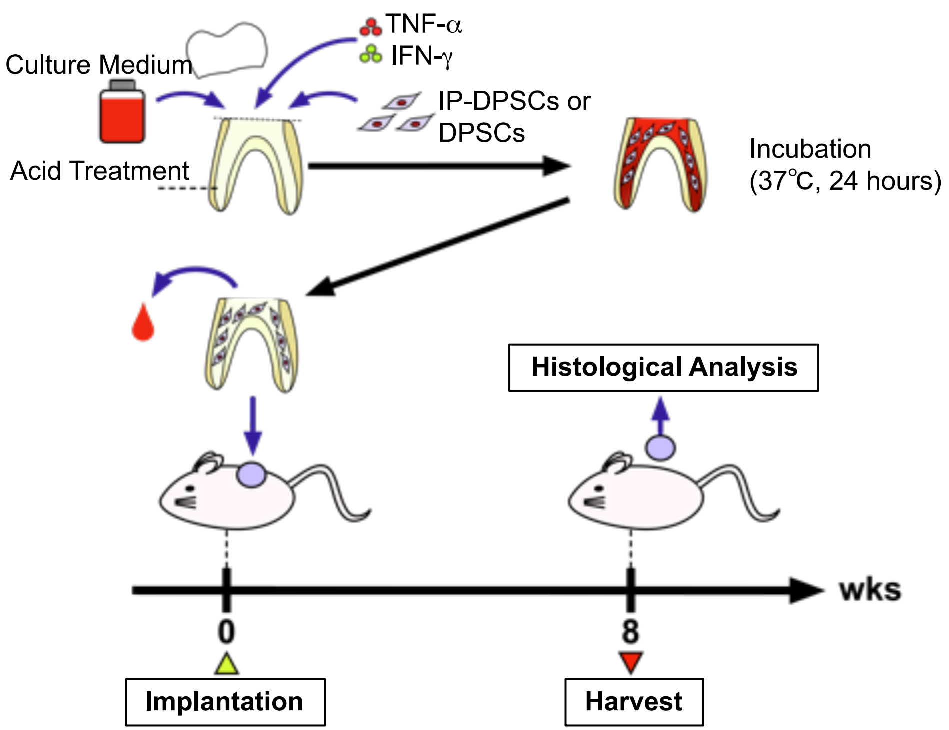

Supplementary Figure 6: A scheme of an assay for *in vivo* dentin regeneration on human dentin of IP-DPSCs. Human teeth were treated with 1% acetic acid, and were loaded IP-DPSCs (2.0x10^6) treated with or without TNF-α (100 ng/ml) or IFN-γ (100 ng/ml) at 37°C for 24 hours. The IP-DPSC-loaded tooth samples were implanted under the dorsal skin of immunocompromised mice, and were harvested for histological analysis eight weeks after the implantation.

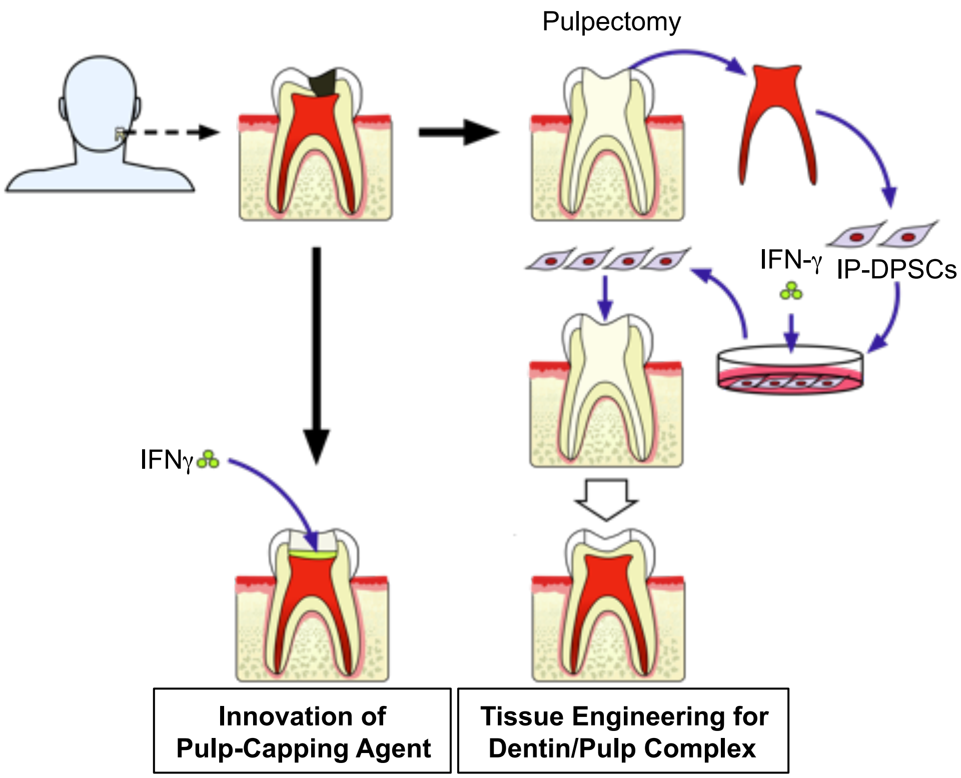

Supplementary Figure 7: A scheme of future regenerative endodontics based on recipient DPSCs accompanied with IFN-γ.
